# Cytochrome P450 Complement May Contribute to Niche Adaptation in *Serpula* Wood-Decay Fungi

**DOI:** 10.3390/jof8030283

**Published:** 2022-03-10

**Authors:** Andrew Cowan, Inger Skrede, Suzy Clare Moody

**Affiliations:** 1 Faculty of Sport, Health and Social Science, Solent University, Southampton SO14 0YN, UK; andrew.cowan@solent.ac.uk; 2 Department of Biosciences, University of Oslo, NO-0316 Oslo, Norway; inger.skrede@ibv.uio.no; 3 Faculty of Science, Engineering and Computing, Kingston University, Kingston-Upon-Thames KT1 2EE, UK

**Keywords:** P450, dry rot, adaptation, *Serpula himantioides*, *Serpula lacrymans* var. *shastensis*, *Serpula lacrymans* var. *lacrymans*

## Abstract

*Serpula* wood-decay fungi occupy a diverse range of natural and man-made ecological niches. *Serpula himantioides* is a forest-floor generalist with global coverage and strong antagonistic ability, while closely related species *Serpula lacrymans* contains specialist sister strains with widely differing ecologies. *Serpula lacrymans* var. *shastensis* is a forest-floor specialist in terms of resource preference and geographic coverage, while *Serpula lacrymans* var. *lacrymans* has successfully invaded the built environment and occupies a building-timber niche. To increase understanding of the cellular machinery required for niche adaptation, a detailed study of the P450 complement of these three strains was undertaken. Cytochrome P450 monooxygenases are present in all fungi and typically seen in high numbers in wood decay species, with putative roles in breakdown of plant extractives and lignocellulose metabolism. Investigating the genomes of these related yet ecologically diverse fungi revealed a high level of concordance in P450 complement, but with key differences in P450 family representation and expression during growth on wood, suggesting P450 proteins may play a role in niche adaptation. Gene expansion of certain key P450 families was noted, further supporting an important role for these proteins during wood decay. The generalist species *S. himantioides* was found to have the most P450 genes with the greatest family diversity and the highest number of P450 protein families expressed during wood decay.

## 1. Introduction

Cytochrome P450 monooxygenases (P450s) are ubiquitous in eukaryotic organisms although the number and composition of the P450 complement varies widely between species, genera and ecological niches [1,2]). Early studies of fungal P450 complement identified Ascomycete saprotrophs *Aspergillus nidulans* and *Neurospora crassa* as having 109 and 41 P450 genes, respectively [3], *A. nidulans* was later suggested to contain 111 P450 genes, [4], while the genome of the model wood decay Basidiomycete *Phanerochaete chrysosporium* was found to contain 148 P450 sequences [5]. Most cytochrome P450s are thought to be adaptive, fulfilling roles that enable the organism to survive in a particular niche or specific environmental conditions. The range of catalytic processes P450 enzymes perform include stereo- and regio-specific epoxidation, dehalogenation and dealkylation, and they are known to be involved in spore wall formation, fatty acid degradation, toxin production and the biotransformation of a variety of aromatic and aliphatic compounds [6]. The presence of numerous P450 proteins in fungal wood decay species appears to be a common feature across both white and brown rot species, with numbers of predicted P450 proteins ranging from 150 to over 300 [1,2,7], with evidence that certain families are expressed during wood decay [8]. A subset of P450 families is represented exclusively in the genomes of fungal wood decay species, and it has been proposed that members of this subset are important for detoxification of plant breakdown products and extractives, potentially increasing the range of nutritional resources available to the fungus [9]. 

The genus *Serpula* includes a range of brown-rot, wood-decay fungi with differing ecological niches. *Serpula lacrymans* var. *lacrymans* (SLL) ((Wulfen) J. Schröt. 1888) causes dry rot in temperate regions by invading the built environment and aggressively decaying structural wood at significant economic cost [10]. Human utilisation of colonised wood in timber constructions is thought to have presented opportunities for this strain to move into the built environment, as it originated in the treelines of temperate and boreal forest, specialising in wood decay [11,12]. *Serpula lacrymans* var. *shastensis* (SHA) ((Harmsen) Ginns and M.N.L. Lefebvre. 1993) and is a closely related but genetically distinct sister strain (see Figure S1 for illustrative tree) which is a treeline, wood-decay specialist but remains geographically restricted to the Cascade mountain range of North America. This strain has not been found in the built environment [13]. A recently published paper [11] compared the genomes of SLL and SHA with *Serpula himantioides* (SH) ((Fr.) P. Karst. 1884), a wood decay fungus widespread in temperate, boreal forest systems, and rarely found in the built environment (although it causes less damage than SLL). Characteristics thought to contribute to the success of SLL in the built environment have been suggested to include the ability to utilise diverse substrates typically thought to be recalcitrant to breakdown, and effective mitigation against plant extractives—functions regularly attributed to cytochrome P450 proteins [14]. In this study, we focussed on characterising the genome P450 complement of these three related *Serpula* strains, analysing their transcriptomic profiles on wood nutrient resources, with a view to understanding P450 evolution, diversity and relevance to ecological niche adaptation.

## 2. Materials and Methods

### 2.1. Cultures and Conditions

The three strains used in this study were *Serpula lacrymans* var. *lacrymans* (SL200) referred to as SLL, *Serpula lacrymans* var. *shastensis* (SHA17-1) referred to as SHA and *Serpula himantioides* (MUCL38935) referred to as SH. These were grown as described in [15], on Czapek Dox (CD) media, supplemented with sucrose or sawdust from *Pinus sylvestris* (pine) or *Picea abies* (spruce). These trees are indicative species of the typical ecological niches (in the natural and built environment) of the strains of interest. 

### 2.2. Analysis of Predicted Cytochrome P450 Genes

Based on the genome annotation reported in [15] lists of predicted P450 genes containing a putative PF00067 domain in the genomes of SLL, SLS and SH were compiled. Amino acid sequences of predicted proteins were subjected to BLAST analysis against the Nelson Cytochrome P450 database via http://bioshell.pl/p450/blast_only.html (last accessed on 28 November 2020) using the default settings. Results of the search are available in the Supplementary Information (Table S1). Identifications with an E score below 10^−3^ were accepted. The Pfam consensus haem-binding domain (FXXGXRXCXG) and the presence of an EXXR motif in the K helix in a predicted protein longer than 300 amino acids was accepted as a possible cytochrome P450 [16]. Shorter sequences or those lacking an EXXR motif and/or haem-binding domain when aligned in Clustal Omega were treated with caution as pseudogenes or partial sequences [17] were not included further in this study. Proteins containing EXXR motifs with non-conventional amino acid construction were included provided the motif aligned with the EXXR motifs in Clustal Omega. Proteins with at least 40% identity to a known, named, P450 were classified as belonging to the same family, those with at least 55% identity as belonging to the same sub-family. Some of the *Serpula* P450s had already been named and assigned to a sub-family and these are reported as such. Deviation from the Pfam haem-binding domain sequence is common, with the axial cysteine the only completely conserved amino acid, and overall primary sequence known to be very variable. Predicted proteins were described as belonging to a ‘novel’ family if they contained both a haem-binding site that conformed to the Pfam PF00067 domain, and the EXXR motif, but did not have at least 40% identity with any known fungal P450. A full synopsis of family/sub-family assignment is available (Table S1) and the raw sequences can be accessed via https://drive.google.com/drive/folders/1ZfELf5Sy6gJyhOvplBrEYU2PjT3kPVCj?usp=sharing (last accessed on 10 January 2022). 

### 2.3. Generation of Sequence Logo Analysis for Characteristic Motifs

Sequence logos were generated for the AGXDTT, EXXR, XXPERX and FXXGXRX- CXG motifs using WebLogo v3.7.4 (http://weblogo.threeplusone.com/create.cgi, last accessed on 10 January 2022) [18]. The sequences of predicted proteins for each species were aligned in Clustal Omega and the relevant motifs then used to generate the WebLogo (raw data shown in Tables S2–S4). The logos give a visual representation of amino acid conservation across the motif (from N to C terminus) with the relative height of the stack increasing with a higher degree of conservation at that position, and the size of the letter within the stacks indicating relative frequency at that position. These data are presented as probability matrices. The stacks per line was set at the default of 40, and the images were exported as PDF files. 

### 2.4. Generation of Phylogenetic Trees

Evolutionary analysis of putative P450s was carried out via the generation of phylogenetic trees. Sequences were aligned using the Clustal Omega function included in the Unipro UGENE software [19] before being exported for tree generation via Molecular Evolutionary Genetics Analysis (MEGA-X) software [20]. As per previous P450 evolutionary analysis [9], phylogenetic trees were constructed using the minimum evolution method [21] (bootstrap value of 500) and evolutionary distances were computed using the Poisson correction method [22], with units being the number of amino acid substitutions per site. The minimum-evolution tree was searched using the close-neighbour-interchange algorithm [23] at a search level of 1 and the neighbour-joining algorithm was used to generate the initial tree [24]. Tree data were submitted to iTOL (Interactive Tree of Life—https://itol.embl.de/, last accessed on 12 May 2021) for annotation purposes [25]. 

### 2.5. Transcriptomic Analysis

The transcriptomic data analysed here were generated by [15] and as such the experimental methods and analytical techniques used are detailed in their paper. The transcriptomic analysis relevant to this work is briefly described here for clarity. Differential expression was determined using EdgeR v.3.16.5 [26] with the GLM approach and the quasi-likelihood F-test, based on an FDR-corrected *p*-value ≤ 0.05 and absolute log fold change greater than 1. This allowed for two contrasts to be assessed with regards to differential gene expression—genes significantly up-regulated on wood compared to the CD control (denoted ‘core wood’), and those significantly up-regulated on one wood type compared to the other (pine-specific and spruce- specific genes). The original data and analysis files are available at dryad link: https://doi.org/10.5061/dryad.4f4qrfj93 and NCBI Bioproject PRJNA 655420, and https://drive.google.com/drive/folders/1ZfELf5Sy6gJyhOvplBrEYU2PjT3kPVCj?usp=sharing (both last accessed on 10 January 2022). 

## 3. Results

### 3.1. Identification, Nomenclature and Diversity of P450 Complement 

**Figure 1:**
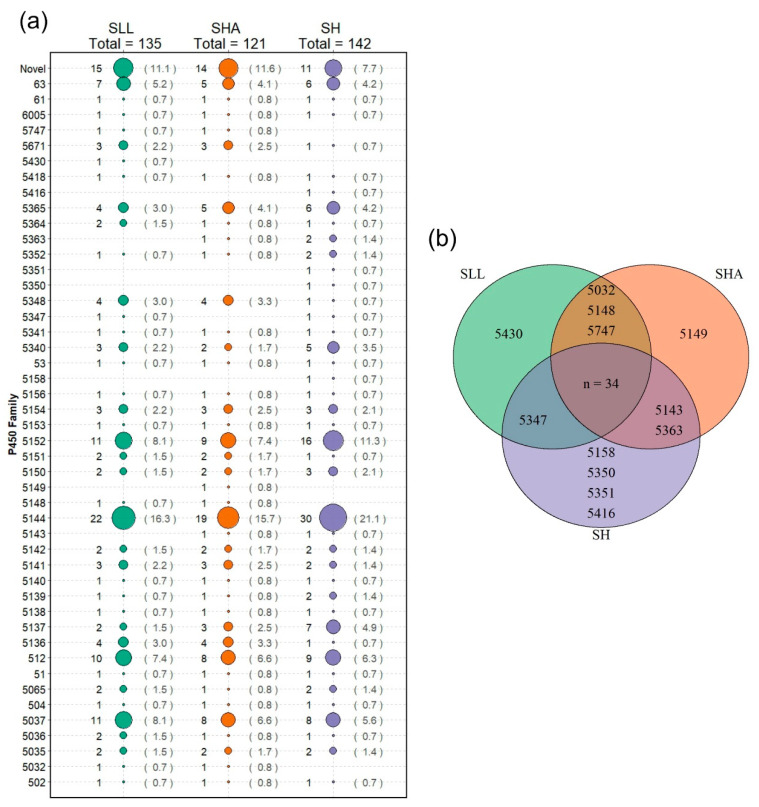
The predicted P450 complement of *S. lacrymans* var. *lacrymans* (SLL), *S. lacrymans* var *shastensis* (SHA) and *S. himantioides* (SH). The suggested family assignment is based on identity of amino acid sequences, with putative P450s showing <40% identity to named P450 sequences described as ‘novel’. (**a**) Bubble size is relative to the number of P450 proteins assigned to that family. For each strain, the number of P450 proteins assigned to the family is on the left of the bubble and percentage of the total P450 count for that strain is given on the right. (**b**) Distribution of P450 families between the three strains. The 34 families shared between all three strains are as follows: CYP51, 53, 61, 63, 504, 512, 5035–5037, 5065, 5136–5142, 5144, 5150–5154, 5156, 5340, 5341, 5348, 5352, 5364, 5365, 5148, 5671, and 6005.

After curation, the genome of SH had 142 predicted functional P450s, SHA had 121 and SLL had 135. These were classified into suggested P450 families based on amino acid identity as per the P450 Nomenclature Committee guidelines [27]. SH had predicted proteins tentatively assigned to 41 different families and 11 proteins which met the strict inclusion criteria but did not have a match greater than 40% with any known P450 protein, which were designated ‘novel’ (Figure 1a). SLL had proteins putatively assigned to 39 families and 15 proteins tentatively identified as novel. Forty families were identified in SHA with 14 probable novel P450 proteins. Families exclusive to one strain were present in all three fungi—SH had representative proteins from families CYP5158, 5350, 5351 and 5416; SHA contained a protein from CYP5149 and SLL had a representative from CYP5430 (Figure 1b). CYP5144 was by far the most enriched family in all three strains. Families 5037, 5152 and 512 were also shown to be enriched with all strains having multiple members, and at least one strain having 10 or more proteins in the family. 

**Figure 2:**
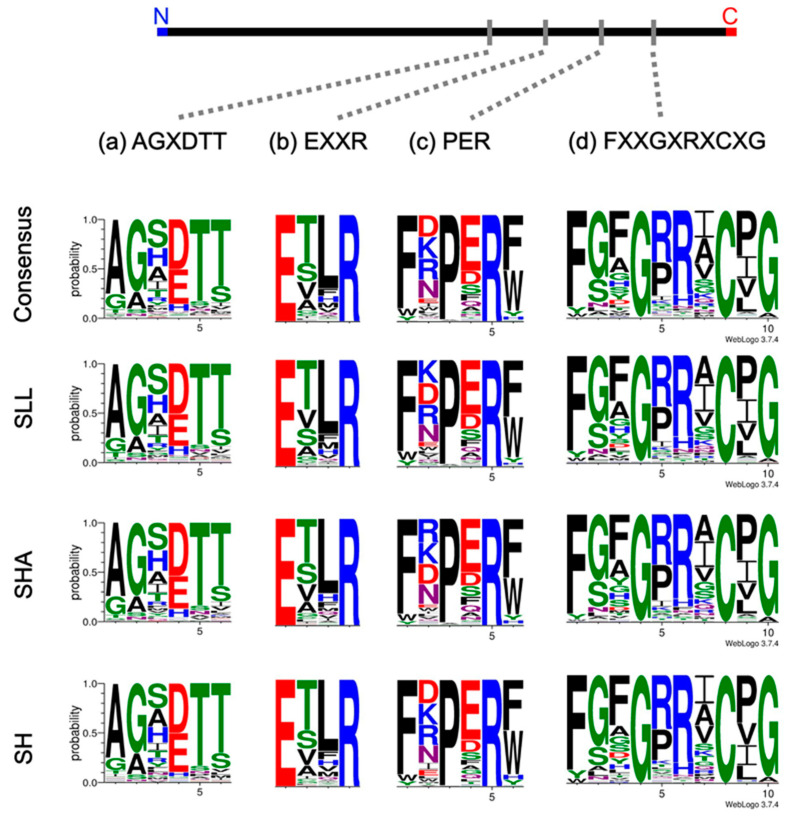
Comparison of the structurally important P450 motifs across the three strains (*S. lacrymans* var. *lacrymans* (SLL), *S. lacrymans* var. *shastensis* (SHA) and *S. himantioides* (SH)) and as a consensus sequence. Relative position of the key structural P450 motifs in the primary sequence is shown at the top with WebLogo [18] probability matrices for those motifs from the three strains are shown below. (**a**) occurs in the I helix, (**b**) occurs in the K helix and together with (**c**) forms the E-R-R triad, and (**d**) is the haem-binding site. The size of the letter is proportionate to the probability of this amino acid occurring in this position. Colour denotes the properties of the amino acid residue—acidic in red, basic in blue, polar in green, hydrophobic in black and neutral in purple.

Four sequence motifs that contribute to P450 protein topology and function are recognised as highly conserved—the haem-binding site FXXGXRXCXG, in which the cysteine functions as a ligand for the haem cofactor; the EXXR motif in the K-helix; AGXDTT located in the I-helix; and PER, which together with EXXR, forms the E-R-R triad. These were compared across all putative P450s in each strain, and as seen in Figure 2, there was a high degree of concordance between strains in all the conserved regions. This was not unexpected as the three strains are closely related and their P450 complements highly similar suggesting the majority of these proteins were present in a common ancestral strain. A full outline of these four sequence motifs for the three *Serpula* is available (Tables S2–S4).

### 3.2. Evidence of P450 Family Expansion Was Seen in All Strains

**Figure 3:**
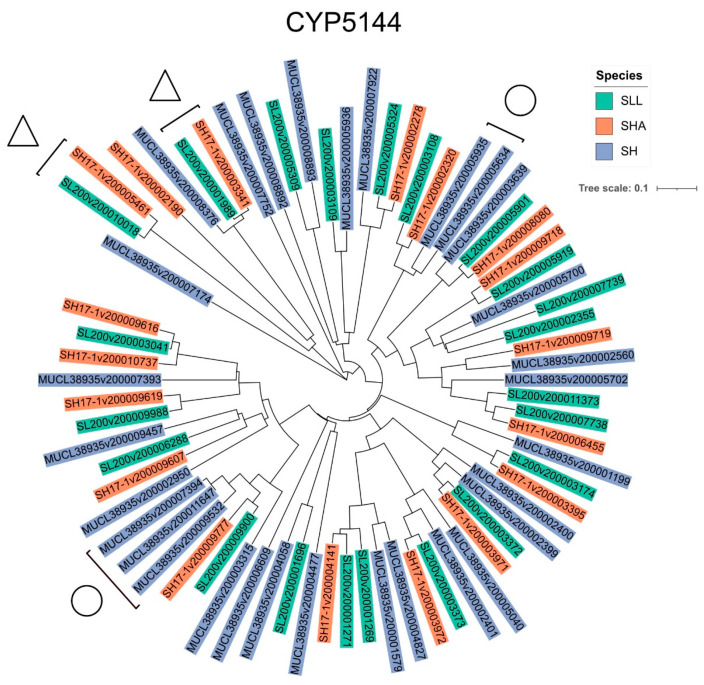
Phylogenetic analysis of putative CYP5144 family members in *S. lacrymans* var. *lacrymans* (SLL), *S. lacrymans* var. *shastensis* (SHA) and *S. himantioides* (SH). A minimum evolution tree for the 71 members assigned to this family was constructed using the close-neighbour-interchange algorithm in MEGA-X [20]. Evidence for gene orthologues highlighted by the triangles and examples of SH strain-specific enrichment in the P450 family through gene duplication events is shown by the circles. Constructs are coloured for each strain for ease of identification—gene prefixes are as follows: SL200 are SLL, SH17 are SHA and MUCL are SH.

The CYP5144 family was significantly enriched in all strains. Members of this family comprised 15–20% of the total P450 complement, with SH possessing the highest number of members (30), followed by SLL (22) and SHA (19). The presence of specific CYP5144 subfamilies was highly consistent between SLL and SHA, with SH showing more variation. Minimum evolution phylogenetic analysis of the 71 putative P450s assigned to this family was carried out and potential orthologous proteins identified (for example, Figure 3, triangles). The resulting tree also suggested evidence for gene-duplication events that may have contributed to the expansion of this family post-speciation (Figure 3, circles), and therefore these P450 proteins are candidates for enabling niche adaptation. P450 families 5037, 5152 and 512 exhibited a level of expansion across the three *Serpula* strains with members contributing >5% of the total P450 complement in each strain. CYP5152 is reported as being the second most prominent family in these three strains. Other families of notable count across the three strains were CYP63 and 5365 (accounting for ≥3% of the total P450 complement in all strains). 

### 3.3. Identification of Novel P450 Sequences Suggested Evidence of Niche Adaptation 

**Figure 4:**
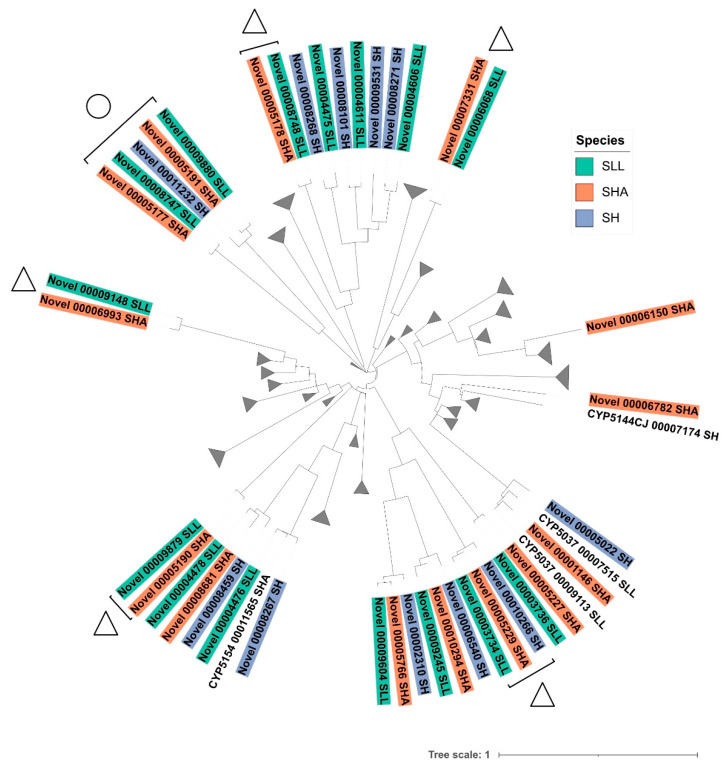
Phylogenetic analysis of the novel P450 sequences identified in *S. lacrymans* var. *lacrymans* (SLL), *S. lacrymans* var. *shastensis* (SHA) and *S. himantioides* (SH). A minimum evolution tree for all 398 sequences was generated using the close-neighbour-interchange algorithm in MEGA-X [20], and clades containing only named P450s were collapsed (represented by the grey triangles). Novel sequences were coloured by species. Evidence for gene orthologues is highlighted by the triangles and a grouping of novel sequences which does not share a branch with any named P450s is highlighted by the circle.

‘Novelty’ was assigned to a protein sequence if it was over 300 amino acids, contained a good match to both EXXR and haem-binding motifs, but had less than 40% identity to any named P450 protein. As stated above, SH contained 11 proteins tentatively identified as novel, SHA 14 and SLL 15. Phylogenetic analysis of these novel sequences highlighted a number of putative orthologous proteins present between SLL and SHA, and to a lesser extent in all three of the *Serpula* strains (Figure 4, triangles). Many of these novel sequences were present within clades containing named P450s (i.e., family/sub- family-assigned sequences). Groupings of novel P450s which do not share a branch with any other known P450 (e.g., Figure 4, circle) are considered more likely to represent proteins with novel catalytic roles or be related to niche-specific functions. 

### 3.4. Differential Expression of Putative P450 Proteins during Growth in Spruce and Pine

**Figure 5:**
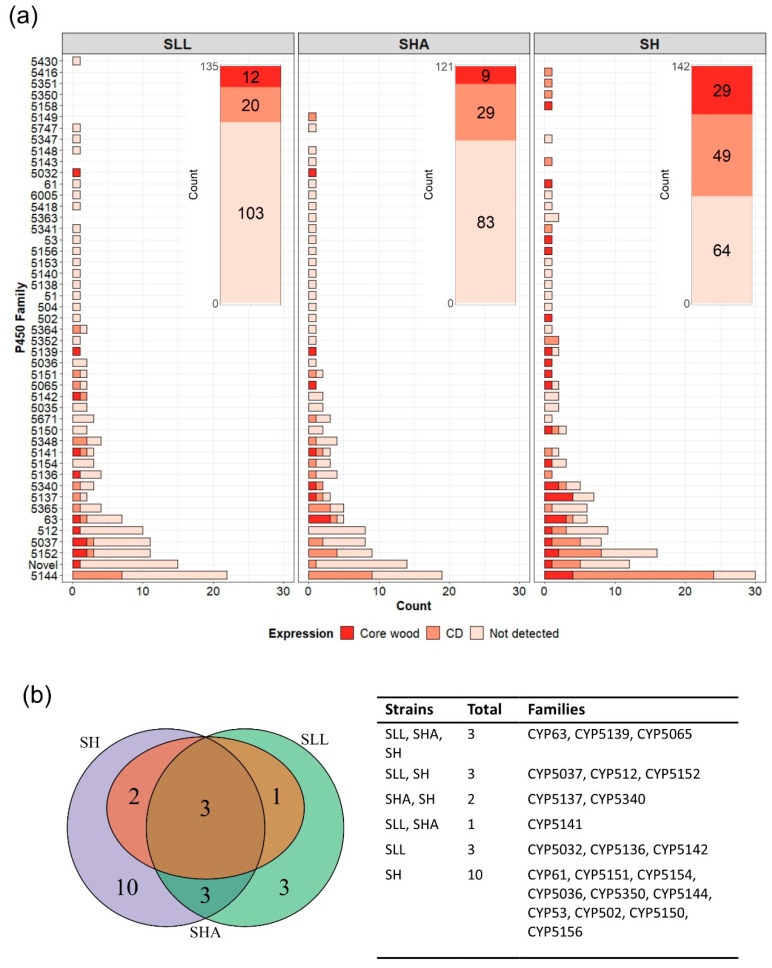
Core wood P450 transcripts for *S. lacrymans* var. *lacrymans* (SLL), *S. lacrymans* var. *shastensis* (SHA) and *S. himantioides* (SH). (**a**) Predicted P450 families that showed significant up-regulation during growth on media supplemented with wood (either pine or spruce) compared to CD agar for each of the *Serpula* strains. P450 families for which expression was not detected under the conditions tested are also shown. The stacked bar insert (top right of each panel) shows the breakdown in terms of the total P450 count falling under each category of expression. (**b**) Venn diagram of the core wood P450 transcripts for each strain with the accompanying table to indicate the P450 families identified.

Ref. [15] investigated the global transcriptomic response of these *Serpula* strains to growth on wood (pine vs. spruce) compared to sucrose-based CD medium. These experimental data were analysed here in greater detail to enhance understanding of which P450s may be important for wood-decay, and the role they may play in niche adaptation when comparing the three strains. Transcripts for all P450 proteins were analysed, and a ‘core wood’ P450 complement was identified by retaining the sequences that corresponded to transcripts significantly up-regulated during growth on both wood types compared to the CD control (Figure 5). 

When interpreting these results, the niche breadth experiments carried out by [15] give some important context as these showed a decomposition of spruce and pine by SH, whereas both *S. lacrymans* variants exhibited a preference for spruce with SHA exhibiting greatly reduced growth on pine. Approximately one third of the core wood P450 complement of SH was exclusive to this strain (Figure 5b), hinting at a significant role of this enzyme superfamily during lignocellulose degradation in this species. The larger number of families represented in core wood transcripts identified in SH (more than double the core wood P450 complement of SLL and more than triple SHA) may reflect its generalist ability to utilise a wider substrate range (e.g., pine, spruce and fir) compared to the other *Serpula* species. The enhanced expression of P450s in SH during wood utilisation is further appreciated when the number of different family members expressed is considered. SH has five families with two or more representatives significantly up-regulated during wood decay, compared with two in SLL and one in SHA (Table S5). Families CYP5137 and 5144 were particularly well-represented in the SH transcriptome. The sister strains of *S. lacrymans* showed clear differences in P450 transcription. While SHA had no P450 families exclusive to its core wood transcriptome, SLL exhibited three families exclusively (CYP5032, 5136 and 5142, Figure 5b), suggesting these may be part of the strain-specific adaptation enabling aggressive utilisation of wood structures. 

**Figure 6:**
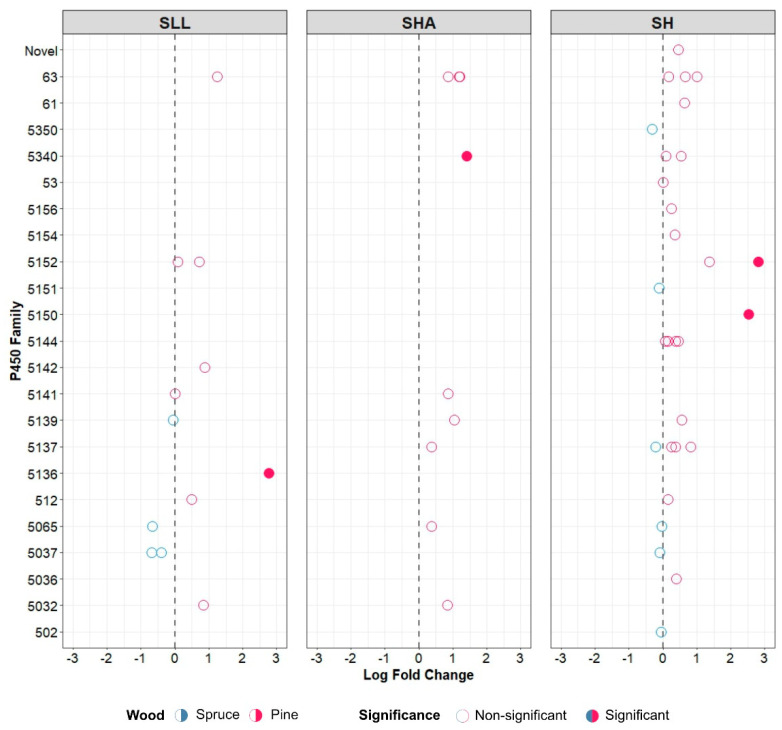
Pine-specific and spruce-specific P450 transcripts for *S. lacrymans* var. *lacrymans* (SLL), *S. lacrymans* var. *shastensis* (SHA) and *S. himantioides* (SH). Predicted P450 families with transcripts differentially up-regulated between growth on media supplemented with pine or spruce are included. A log fold change < 0 was related to expression on media supplemented with spruce, and a log fold change > 0 to media supplemented with pine. Solid points indicate families with significant differential expression between the wood-supplemented media types (*p* ≤ 0.05).

The core wood group of transcripts was further characterised into pine- and spruce-specific P450s based upon their differential expression, with emphasis placed upon those which showed a statistically significant difference in expression between the wood types (Figure 6). 

Few transcripts showed statistically significant up-regulation on either wood compared with the other, although small changes in expression are not necessarily insignificant in biological terms. As seen in Figure 5a, SLL had a core wood transcriptome of 12 families up-regulated on both pine and spruce compared with CD agar, and SHA had 9. SH had 29 P450 families up-regulated during growth on wood, with 10 families exclusively represented compared with SLL and SHA (Figure 5b) and two transcripts (one each from families 5150 and 5152) significantly up-regulated on pine compared with spruce (Figure 6). 

## 4. Discussion

SH occupies a generalist forest floor niche where it is able to utilise a range of softwoods [28], while *S. lacrymans* has evolved to occupy more specialist environments. While SHA is both geographically restricted and a specialist for colonisation of *Abies magnifica* [13], the sister strain SLL has adapted to the indoor environment, specialising in decay of structural timber. Genomic analysis suggested that SLL may have been pre-adapted to the built environment and therefore colonised and thrived in the new niche without needing extensive adaptation to survive [11,12]. This work investigated the P450 complement of these three *Serpula* strains and the role these important proteins may play in niche adaptation. The large P450 complement of wood-decay fungi has drawn interest over the last decades as genome sequencing has revealed the commonality of this feature, with P450 proteins thought to play key roles in detoxification of lignocellulose breakdown products and potentially expanding the range of resources the fungus is able to metabolise [9]. The size of the *Serpula* P450 complement correlates well with early studies of both Ascomycete and Basidiomycete genomes [3,5], suggesting P450-driven metabolism is a key fungal adaptation. The variation in which P450 families are present in a given species can hint at further P450-enabled niche adaptation. The functionality of the P450 complement is supported in each *Serpula* strain by at least two cytochrome P450 reductases (EC1.6.2.4) encoded in the genome (a similar number to other wood-decay fungi), although differential expression of these genes was not noted in the experimental conditions used. 

SH was found to have the largest predicted P450 protein complement, with four families unseen in the other two strains (CYP5158, 5350, 5351 and 5416), and also by far the most P450 families represented in the transcriptome on wood. With the ecological role of SH as a forest-floor saprotroph, capable of utilising a wide range of resources, the expansion of P450 proteins suggests enhanced catalytic versatility to enable survival in a heterogeneous nutrient environment [29]. SLL contained a higher number of putative P450 proteins compared with sister strain SHA, with each strain having one family exclusively present in the genome. This suggests the increase in total P450 proteins in SLL is a likely result of strain-specific gene expansion. The colonisation of indoor timber presents some challenges compared with fallen wood. Plant extractives produced to prevent fungal colonisation [30] are typically higher in timber than in partially decayed wood on the forest floor and can be augmented through treatment with biocide compounds [31]. The xenobiotic breakdown of such compounds is thought to be mediated by P450 enzymes [30], so the expansion of the SLL P450 complement compared with SHA may reflect adaptation to enable this. P450 proteins are not the only family of enzymes expanded in SLL to enable niche adaptation as proteins for rapid hyphal expansion, effective nutrient and water translocation, and expanded nutrient capture have all been previously identified as playing a role in this [11,32]. In contrast to SLL, SHA is a specialist, both in terms of nutrient preference and geography. This niche adaptation may have fostered a more streamlined genome, with little requirement to expand catalytic capacity [33]. 

As expected in closely related organisms, the P450 sequence motifs showed a high level of conservation across the three *Serpula* strains. The FXXGXRXCXG motif was well-conserved across all three strains, with proline the most common (42.5% of the predicted P450 sequences) in the CXG motif. The EXXR motif was also well-conserved with both glutamic acid (E) and arginine (R) appearing completely conserved in SLL and SHA. One divergent EXXR motif was seen in SH, with isoleucine seen as a radical change in the carboxy terminal position. ETLR (threonine and leucine) was the most common sequence in all strains (38.5 % of the predicted P450 sequences). Some family-specific characteristics were identified in this study including a preference towards EVLR in CYP5037, and the glutamine present in the EXXR of CYP512 together with the histidine and alanine present in positions seven and eight of the haem-binding sequence. The double arginine characteristic present within the haem-binding motif of families 5144, 5152 and 5037 was also observed. These findings are in agreement with previous work comparing a selection of basidiomycetes [16]. Novel sequences, with less than 40% identity to named P450s, were identified in all strains. Many of these cluster together in the phylogenetic tree (Figure 4) suggesting orthologues are present in at least SLL and SHA, with some possible orthologous sequences conserved between all three strains suggesting a common ancestry of these putative proteins. 

CYP5144 was the most enriched family in the strains studied, followed by 5152, 5037, 512 and 63, which concurs with previous work [9]. Of these families, CYP5144, 512, 5037 and 63 have all previously shown enhanced expression during fungal growth on wood [1,14,34] and several of these are represented by more than one protein per family in the core wood transcriptomes. While the exact ecological role of many fungal P450 proteins remains cryptic, there is evidence for understanding the catalytic activity for some of these families. CYP512 proteins demonstrate activity towards dehydroabietic acid [2]—a component of the protective resin produced by trees of temperate coniferous forests as a preservative against microbial attack [35]. Each of the *Serpula* strains contained at least 15 proteins tentatively assigned to CYP5144 (Figure 1), a family known to be involved in the oxidation of polycyclic aromatic hydrocarbons and steroids, and the biosynthesis of triterpenoids [36,37,38] and which may play an important role in xenobiotic breakdown of plant extractives or degradation of lignocellulose per se. Their importance in some aspect of wood decay is strongly suggested as they are one of the few enriched families identified by [9] and have yet to be found in species with alternative ecological niches. Five CYP5144 proteins were present in the SH core wood transcriptome, suggesting family expansion and potentially diversification of function for niche adaptation. The importance of these families in adaptation to the wood-decay niche can be appreciated through comparison with other filamentous fungi. A key study [3] of P450 genes in the filamentous fungal saprophytes *A. nidulans, N. crassa*, and plant pathogens *Fusarium graminearum* and *Magnaporthe grisea* found none of the P450 families enriched across the wood decay basidiomycetes and none of the families found exclusively in the strains in this study.

The transcriptome revealed families CYP63, 5139 and 5065 as core wood transcripts common to all three strains suggesting their involvement in fundamental aspects of wood-decay. Three CYP63 members were seen significantly up-regulated in the core wood transcriptomes of both SHA and SH, perhaps suggesting forest floor functionality—previous studies [9,39] identified CYP63 as one of the enriched families in basidiomycetous fungi, with high functional diversity suggested across both brown and white rot fungi [2,39,40] The paucity of functional studies on P450 proteins means the possible function of the other two families remains unknown. Of the spruce- and pine-specific transcripts identified, a small number of pine-specific P450s were deemed as showing a significant difference in expression between the wood types. This increased expression of P450s on pine may reflect the high levels of extractives (e.g., resin acids) found in this wood type [41]. CYP5150 which was found to be a pine-specific P450 in SLL in the present study, has been previously associated with acting on dehydroabietic acids and stilbenes when profiled in *P. placenta* [2]. Only one of the P450 families per *S. lacrymans* strain showed significant changes in expression between the wood types (Figure 6, CYP5340 for SHA and CYP5136 for SLL both up-regulated on pine), suggesting a general ‘wood utilisation P450 toolbox’ for this species and a very limited number of particular P450 proteins being used for specific woody substrates. Of note, CYP5136 is one of the three families exclusively expressed by SLL compared with the other strains. 

While the P450 complement of all three strains initially appears highly similar, the differences seen in genomic P450 diversity and core wood transcriptome may reflect the divergence of these strains in regard to their substrate preferences, habitat and combative ability [15]. Of particular interest is the higher number of P450 families represented in the SH genome, and the higher number of P450s expressed by this species on wood. This species is more generalist compared with the *S. lacrymans* and able to effectively compete for a range of resources in forest habitats around the globe, and perhaps the wider catalytic ability enabled by P450 proteins facilitates this ubiquitous distribution. In contrast, SLL and SHA have slightly smaller P450 complements with both sub-species having a member of a P450 family that is unrepresented in the genomes of the other two strains. This suggests an ancestral P450 complement for general wood utilisation, followed by strain-specific gene expansion to enable successful niche specialisation. 

## Data Availability

The original data and analysis files are available at dryad link: https://doi.org/10.5061/dryad.4f4qrfj93 and NCBI Bioproject PRJNA 655420, and https://drive.google.com/drive/folders/1ZfELf5Sy6gJyhOvplBrEYU2PjT3kPVCj?usp=sharing (both last accessed on 10 January 2022).

## Supplementary Materials

The following supporting information can be downloaded at: https://www.mdpi.com/article/10.3390/jof8030283/s1, Figure S1: A simplified sketch of the phylogenetic relationships of *Serpula himantioides, S. lacrymans* var. *lacrymans* and *S. lacrymans* var. *shastensis*, and their placement in the Serpulaceae, based on phylogenies and molecular dating in [42]); Table S1: Classification of predicted P450s for each of the three *Serpula* species. Family/subfamily nomenclature was assigned as per the P450 Nomenclature Committee Guidelines [27]; Table S2: Sequence motifs for the putative P450s identified in *Serpula lacrymans* var. *lacrymans* (SLL) SL200. Multiple sequence alignment was carried out using Clustal Omega and sequence position (indicating the position of the first aa) is given alongside. Dashes are used to indicate absence/incomplete region of a sequence; Table S3: Sequence motifs for the putative P450s identified in *Serpula lacrymans* var. *shastensis* (SHA) SHA-17. Multiple sequence alignment was carried out using Clustal Omega and sequence position (indicating the position of the first aa) is given alongside. Dashes are used to indicate absence/incomplete region of a sequence; Table S4: Sequence motifs for the putative P450s identified in *Serpula himantioides* (SH) MUCL38935. Multiple sequence alignment was carried out using Clustal Omega and sequence position (indicating the position of the first aa) is given alongside. Dashes are used to indicate absence/incomplete region of a sequence. * indicates the two halves of the large fusion protein MUCL38935v200007922; Table S5: The number of significantly up-regulated family members seen in the core wood transcriptome in each strain.
