# Supplementary material for: Cytochrome P450 Complement May Contribute to Niche Adaptation in Serpula Wood-Decay Fungi
Source: J Fungi (Basel). 2022 Mar 10;8(3):283. doi: 10.3390/jof8030283 (PMC8949155; doi:10.3390/jof8030283)
Supplement: Supplementary file 1 [file jof-08-00283-s001.zip › jof-1583673-supplementary.pdf]

Supplementary Information for:

Cytochrome P450 complement may contribute to niche adaptation in *Serpula* wood decay fungi, Cowan RC, Skrede I and Moody SC. Corresponding author: SC Moody, Kingston University, s.c.moody@kingston.ac.uk

Figure S1. A simplified sketch of the phylogenetic relationships of *Serpula himantioides, S. lacrymans* var. *lacrymans* and *S. lacrymans* var. *shastensis*, and their placement in the Serpulaceae, based on phylogenies and molecular dating in Skrede et al. (2011).

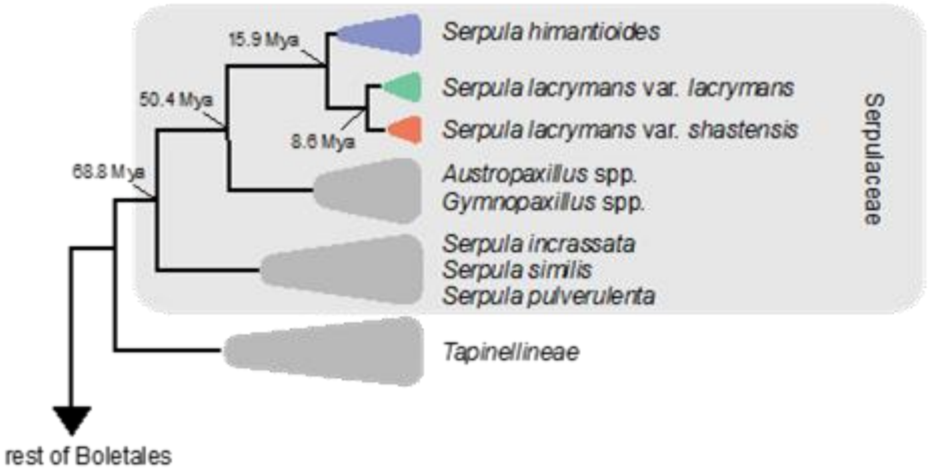

Table S1. Classification of predicted P450s for each of the three *Serpula* species. Family/subfamily nomenclature was assigned as per the P450 Nomenclature Committee Guidelines (Nelson, 2006).

Family (subfamily)	Species		
	Serpula lacrymans var. lacrymans (SLL)	*Serpula lacrymans var. shastensis* (SHA)	*Serpula himantoides* (SH)
502	1 (A: 1)	1 (A: 1)	1 (A: 1)
5032	1 (B: 1)	1 (B: 1)	0
5035	2 (AF: 2)	2 (AF: 2)	2 (AF: 2)
5036	2 (N/A: 2)	1 (N/A: 1)	1 (N/A: 1)
5037	11 (B: 8, N/A: 3)	8 (B: 7, N/A: 1)	8 (B: 8)
504	1 (N/A: 1)	1 (N/A: 1)	1 (N/A: 1)
5065	2 (B: 1, NS: 1)	1 (B: 1)	2 (B: 1, NS: 1)
51	1 (F: 1)	1 (F: 1)	1 (F: 1)
512	10 (AV: 5, AW: 1, N/A: 4)	8 (AV: 6, AW: 1, N/A: 1)	9 (AV: 5, AW: 1, N/A: 3)
5136	4 (L: 4)	4 (L: 4)	1 (N/A: 1)
5137	2 (A: 1, F: 1)	3 (A: 1, F: 1, N/A: 1)	7 (A: 1, F: 5, N/A: 1)
5138	1 (A: 1)	1 (A: 1)	1 (A: 1)
5139	1 (D: 1)	1 (D: 1)	2 (D: 1, NS: 1)
5140	1 (A: 1)	1 (A: 1)	1 (A: 1)
5141	3 (A: 3)	3 (A: 3)	2 (A: 2)
5142	2 (N/A: 2)	2 (N/A: 2)	2 (N/A: 2)
5143	0	1 (N/A: 1)	1 (N/A: 1)
5144	22 (BW: 11, BX: 1, C: 1, N/A: 9)	19 (BW: 5, BX: 1, C: 1, N/A: 12)	30 (BW: 10, BX: 1, CJ: 1, DZ: 1, N/A: 17)

5148	1<br>(A: 1)	1<br>(A: 1)	0
5149	0	1<br>(N/A: 1)	0
5150	2<br>(AG: 2)	2<br>(AG: 2)	3<br>(AG: 2, N/A: 1)
5151	2<br>(A: 1, E: 1)	2<br>(A: 1, E: 1)	1<br>(E: 1)
5152	11<br>(A: 1, G: 6, H: 2, N/A: 2)	9<br>(A: 1, G: 5, H: 2, N/A: 1)	16<br>(G: 8, H: 6, N/A: 2)
5153	1<br>(B: 1)	1<br>(B: 1)	1<br>(B: 1)
5154	3<br>(H: 1, N/A: 2)	3<br>(H: 1, N/A: 2)	3<br>(H: 2, N/A: 1)
5156	1<br>(A: 1)	1<br>(A: 1)	1<br>(A: 1)
5158	0	0	1<br>(N/A: 1)
53	1<br>(C: 1)	1<br>(C: 1)	1<br>(C: 1)
5340	3<br>(A: 3)	2<br>(A: 2)	5<br>(A: 5)
5341	1<br>(B: 1)	1<br>(B: 1)	1<br>(N/A: 1)
5347	1<br>(N/A: 1)	0	1<br>(N/A: 1)
5348	4<br>(N/A: 4)	4<br>(N/A: 4)	1<br>(N/A: 1)
5350	0	0	1<br>(N/A: 1)
5351	0	0	1<br>(N/A: 1)
5352	1<br>(A: 1)	1<br>(A: 1)	2<br>(A: 2)
5363	0	1<br>(N/A: 1)	2<br>(N/A: 2)
5364	2<br>(N/A: 2)	1<br>(N/A: 1)	1<br>(N/A: 1)
5365	4<br>(C: 2, N/A: 2)	5<br>(C: 2, N/A: 3)	6<br>(C: 5, N/A: 1)
5416	0	0	1<br>(F: 1)
5418	1<br>(B: 1)	1<br>(B: 1)	1<br>(B: 1)

5430	1<br>(N/A: 1)	0	0
5671	3<br>(B: 3)	3<br>(B: 3)	1<br>(B: 1)
5747	1<br>(A: 1)	1<br>(A: 1)	0
6005	1<br>(A: 1)	1<br>(A: 1)	1<br>(A: 1)
61	1<br>(A: 1)	1<br>(A: 1)	1<br>(A: 1)
63	7<br>(A: 6, B: 1)	5<br>(A: 5)	6<br>(A: 5, B: 1)
Novel	15	14	11
Number of families	39	40	42
Number of subfamilies	40	38	35
Total number of P450s	135	121	142

Table S2. Sequence motifs for the putative P450s identified in *Serpula lacrymans* var. *lacrymans* (SLL) SL200. Multiple sequence alignment was carried out using Clustal Omega and sequence position (indicating the position of the first aa) is given alongside. Dashes are used to indicate absence/ incomplete region of a sequence.

**Sequence**	**AGXDTT**	**EXXR**	**PER**	**FXXGXRXCXG**	**Family**
SL200v200000479	AGTDTV 300	ESLR 358	FRPERW 414	FSRGPRSCIG 434	CYP5142
SL200v200000480	GGSDTT 298	EILR 355	FNPERF 407	FGYGRRICPG 429	CYP5037
SL200v200000535	AGHETS 348	ETLR 411	FKPERF 482	FSAGPRACIG 503	CYP5671
SL200v200000541	GGSHST 314	EVLR 371	FNPDRF 424	FGAGRRICPG 453	CYP5148
SL200v200000542	AGSDTQ 294	EVLR 351	FNPQRY 404	FGFGRRACPG 420	CYP5747
SL200v200000550	——	EGVR 400	YRPSRW 465	HPTGPRACIG 539	CYP5154
SL200v200000553	AGHETT 454	EGLR 510	YRPSRW 575	FSVGPRACIG 611	CYP5154
SL200v200000615	AGQHTS 322	ETLR 383	WDPSRW 445	FGAGRHRCIG 490	CYP51
SL200v200000639	ISIQVL 294	ESLR 350	FDPSRF 404	FGHGKNACPG 433	CYP512
SL200v200000824	AAIHTS 296	EIQR 352	——	FGHGRHACPG 404	CYP512
SL200v200001221	AGADTT 194	EVMR 251	FKPERF 304	FGYGRRICPG 323	CYP5037
SL200v200001226	GGTDTT 318	EVVR 375	FKPERF 427	FGFGRRICPD 446	CYP5037
SL200v200001269	AASDTT 309	ETLR 366	FKPERF 419	FGFGRRVCIG 439	CYP5144
SL200v200001271	AASETT 334	ETLR 391	FKPERF 442	FGFGRRVCIG 462	CYP5144
SL200v200001362	AGHETT 170	ETLR 226	FRPERW 293	FLSGSRGCIG 318	CYP5138
SL200v200001695	AGAETT 296	EVMR 353	FKPE－ 406	——－RICPG 410	CYP5037
SL200v200001696	AGSDTT 301	ETLR 358	FKPERF 411	FGFGRRICVG 432	CYP5144
SL200v200001989	AGAESE 295	EVLR 352	FWPERF 405	FGYGRRICPG 429	CYP5144
SL200v200002015	ASIHQT 296	EVMR 358	FRPERW 411	FGLGRWACPG 434	CYP5347
SL200v200002128	AGRDTT 363	ETLR 420	FDPDRF 480	FNAGPRICLG 504	CYP63
SL200v200002158	SAIHTS 296	ETLR 352	FDPFRF 406	FGHGKNACPG 435	CYP512
SL200v200002355	AASEMT 282	ETFR 339	FKPERF 392	FGFGRRVCIG 413	CYP5144
SL200v200002605	AGHETT 321	ETLR 378	FKPSRF 439	FSAGHRGCVG 456	CYP5141
SL200v200002606	AGHETT 321	ETLR 378	FKPSRF 439	FSAGYRGCVG 456	CYP5141
SL200v200002613	AGHETT 322	EALR 379	FKPSRF 440	FSAGHRGCVG 457	CYP5141
SL200v200002698	AGSDTT 293	ESLR 350	FEPQRW 403	FGFGRRVCPG 424	CYP5152
SL200v200002699	AGSDTT 295	ESFR 350	FQPERW 405	YGFGRRVCAG 426	CYP5152
SL200v200002700	AGSDTT 295	ETFR 352	FQPERW 405	YGFGRRVCVG 426	CYP5152
SL200v200002987	AGRDTT 366	ETLR 423	FDPDRF 483	FNAGPRICLG 507	CYP63
SL200v200003041	GAAETT 233	ESHR 290	FVPERF 343	FGFGRRVCAG 364	CYP5144
SL200v200003050	AGSDTT 293	ESFR 350	FEPQRW 403	YGFGRRACPG 424	CYP5152
SL200v200003108	AAAETS 302	ETLR 359	FKPERF 423	FGFGRRICVG 433	CYP5144

SL200v200003109	GGAETT 155	ETLR 212	FKPERF 265	FGFGRRICPG 286	CYP5144
SL200v200003174	AGSETT 314	ETLR 371	FKPERF 424	YGFGRRICVG 445	CYP5144
SL200v200003180	AGADTI 374	ESFR 431	FNPLRW 484	YGFGQRACPG 505	CYP5152
SL200v200003187	AGTDTT 325	EVFR 384	FMPERF 437	FGFGRRKCPG 459	CYP5065
SL200v200003372	AAAETT 304	ETLR 361	FKPERF 414	FGFGRRICVG 434	CYP5144
SL200v200003373	AAAETT 302	ETLR 359	FEPDRF 412	FGFGRRICVG 432	CYP5144
SL200v200003734	ANIDVT 326	ESAR 384	FRPERF 438	YGLGPRQCLG 458	Novel
SL200v200003736	ANVDVS 315	ETLR 369	FKPERF 424	FGFGPRQCLG 444	Novel
SL200v200003795	GGADTT 298	EVLR 355	FDPERF 408	FGFGRRICPG 430	CYP5037
SL200v200003820	AGSDTL 304	ETLR 362	FAPERW 416	FSKGPRGCLG 436	CYP5142
SL200v200003844	AAMDTT 326	ETLR 383	WKPERW 450	FLGGGRACIG 478	CYP5150
SL200v200003848	AAMDTT 325	ETLR 382	WKPERW 449	FLGGGRSCIG 477	CYP5150
SL200v200004057	GGMDTN 297	EVFR 354	FSPERY 407	FGFGRRVCPG 427	CYP5037
SL200v200004363	AGSDTT 426	EALR 485	FRPERW 540	FSFGPRACVG 560	CYP53
SL200v200004475	IGTAPS 302	ETVR 372	WNPARW 424	WGIGMHPCLG 447	Novel
SL200v200004476	AGHETT 316	EAMR 373	FEPSRW 436	FGHGPRVCIG 454	Novel
SL200v200004478	AGHETS 320	ETMR 377	FEPSRW 440	FSHGTRACIG 458	Novel
SL200v200004519	AGHETT 318	ETLR 380	YRPERW 447	FGSGPHACIG 474	CYP5430
SL200v200004605	TANSDS 282	ETLR 338	FEPFRF 392	FGLGRHACPG 421	CYP512
SL200v200004606	IGTNTT 288	ETVR 358	WDPARW 410	WGAGLHPCHG 433	Novel
SL200v200004611	TGTITT 288	ETNR 358	WDPSRW 410	WGVGLHPCLG 433	Novel
SL200v200004878	GAIHVC 332	ETLR 410	FDPFRF 483	FGHRRSACPG 512	CYP512
SL200v200005082	AGHETT 352	ESLR 409	FRPERW 462	FIAGPHACIG 483	CYP5032
SL200v200005092	AGRDTT 363	ETLR 420	FDPDRF 480	FNAGPRICLG 504	CYP63
SL200v200005229	AGHETS 350	ETLR 413	FKPARF 484	FSAGPRGCIG 505	CYP5671
SL200v200005309	AGTGTT 303	ELFR 360	FHPERH 413	FGFGRRICPG 434	CYP5144
SL200v200005324	AGFETT 309	ETMR 366	FLPERH 419	FGFGRRICPG 442	CYP5144
SL200v200005509	VGHETT 316	EGLR 370	FKPERW 437	FCDGPRMCIG 464	CYP5341
SL200v200005511	AGHETS 349	ETLR 411	FKPARF 482	FSGGPRSCIG 503	CYP5671
SL200v200005559	AGSDTI 303	EVHR 360	FRPERY 414	FGFGRRLCPG 437	CYP5348
SL200v200005560	AGSDTI 305	EVHR 362	FRPERF 416	FGFGRRICPG 439	CYP5348
SL200v200005646	AGSDTV 275	EVHR 332	FRPERF 372	FGFGRRVCPG 395	CYP5348
SL200v200005647	AGSDTI 306	EVHR 363	FRPERF 417	FGFGRRACPG 440	CYP5348
SL200v200005714	AGHDTT 307	EALR 366	FRPERF 419	FNGGPRRCPA 440	CYP5365
SL200v200005716	AGHDTT 294	ETLR 353	FRPERF 406	FSTGPRQCPA 427	CYP5365
SL200v200005717	AGHETT 289	ESLR 348	FRPERF 401	FSSGPRQCPA 421	CYP5365

SL200v200005784	ASQDAM 311	ESMR 370	LSPERW 423	FGSGPHKCIG 445	CYP61
SL200v200005796	AGSDST 347	ETLR 408	FNPDRW 478	FSFGPHACPG 504	CYP5151
SL200v200005855	TAIHSS 297	ETLR 353	FNPFRF 407	FGHGRGSCPG 436	CYP512
SL200v200005882	AGHETT 327	ETLR 382	FKPERW 449	FLGGPRACIG 474	CYP5139
SL200v200005901	AASESS 305	ETLR 362	FRPERF 415	FGFGRRNCVG 436	CYP5144
SL200v200005919	AASETS 302	ETLR 359	FKPERF 412	FGFGRRVCVG 433	CYP5144
SL200v200005965	AGGDTT 370	ETLR 427	FDPDRF 486	FNAGPRICLG 510	CYP63
SL200v200005966	AGRDTT 367	ETLR 424	FDPDRF 483	FNAGPRICLG 507	CYP63
SL200v200006063	——	EVLR 263	FDPMRY 318	FGYGRLKCVA 335	CYP5153
SL200v200006068	ATIHTL 321	ESLR 377	FDGFRF 431	FGYGSLACPG 457	Novel
SL200v200006288	GGSETT 304	ESFR 361	FKPERF 414	FGFGRRICVG 435	CYP5144
SL200v200006340	AGHETT 397	EVLR 459	FDPERW 526	FSGGIRSCIG 552	CYP5137
SL200v200006406	AGYETT 330	EVLR 385	FNPERW 452	FSDGPRTCLG 479	CYP5340
SL200v200006407	AGYETT 332	ETLR 388	FNPDRW 455	FADGPRTCLG 482	CYP5340
SL200v200006776	AGSDTT 337	EVLR 403	FDPDRW 472	FSAGPRSCIG 497	CYP5151
SL200v200006919	GGSDTT 298	EVLR 355	FNPDRF 408	FGFGRRICPG 430	CYP5037
SL200v200006949	AGARAT 293	ETLR 367	FDPGRK 419	WGGGKTICLG 436	CYP5156
SL200v200007054	ASIDTT 305	ECLR 362	FKPERY 415	FGFGRRRCPG 440	CYP502
SL200v200007149	GGADTT 299	EVLR 356	FDPSRF 409	FGFGRRICPG 431	CYP5037
SL200v200007165	AGTDTM 315	EGLR 373	FLPDRW 436	FGMGSRACGG 461	CYP5140
SL200v200007241	AAIHTS 296	EAQR 352	FQPFRF 406	FGHGRHACPG 436	CYP512
SL200v200007306	TAIHTS 330	ETLR 386	FVPFRF 440	FGHGRNACPG 469	CYP512
SL200v200007502	AGHETT 352	EGVR 408	YRPSRW 473	FSVGPRACIG 503	CYP5154
SL200v200007515	GGSDTT 180	ECMR 237	FDPVRF 290	YGFGRRICPG 320	CYP5037
SL200v200007696	AGSDTS 296	ETLR 353	FNPARW 406	FGFGRRSCPG 427	CYP5152
SL200v200007697	AGSDTT 404	ETLR 461	FNPQRW 514	FGFGRRACPG 535	CYP5152
SL200v200007738	SGSETS 327	ETLR 384	YKPERF 437	FGFGRRICVG 458	CYP5144
SL200v200007739	AASETS 274	ETLR 331	FKPERF 384	FGFGRRICVG 405	CYP5144
SL200v200007879	AAIHTS 344	EVLR 400	FDPFRF 454	FGYGRHACPG 484	CYP512
SL200v200007939	AGSDTT 297	ESFR 354	FNPRRW 407	FGFGRRACPG 428	CYP5152
SL200v200007997	GGADTI 299	EVLR 355	FKPERH 408	FGFGRRICPG 428	CYP5352
SL200v200008105	——	EALR 924	FDASRS 976	RGDGAFKCLG 988	CYP6005
SL200v200008363	GGSDNT 350	ETLR 408	FLPERW 461	FSSGPHGCIG 485	CYP5364
SL200v200008482	AARDTT 364	ETLR 420	FDPERW 486	FSAGPRICIG 510	CYP63
SL200v200008715	AGHETT 315	ESLR 377	WNPNRF 444	FSAGIRACIG 469	CYP5136
SL200v200008723	AGHDTT 315	ESLR 377	WNPNRF 444	FSAGIRACIG 469	CYP5136

SL200v200008747	ALTSNT 285	ESLR 354	FVATRF 417	FGGGISVCSG 436	Novel
SL200v200008748	AGSVNT 303	ETIR 371	WNPNRW 423	WGVARFPCTG 444	Novel
SL200v200008750	AGRDTT 363	ETLR 420	FDPDRF 480	FNAGPRICLG 504	CYP63
SL200v200008769	AGHDTT 315	ETLR 377	WNPNRF 444	FSAGIRACIG 469	CYP5136
SL200v200008771	AGSDTT 416	EALR 475	FWPERW 532	FSFGPAMCVG 570	CYP5035
SL200v200008773	AGSDTT 343	ETLR 401	FWPERW 458	FSFGPANCVG 495	CYP5035
SL200v200008921	AGADTT 294	ETLR 351	FDPQRW 404	FGFGRRVCPG 425	CYP5152
SL200v200008922	AGFDTT 293	EALR 350	FDPGRW 403	FGFGRRVCPG 424	CYP5152
SL200v200009113	GGSDTT 140	ECMR 197	FDPVRF 250	YGFGRRICPG 280	CYP5037
SL200v200009122	AGYEPV 306	ETIR 360	FRPERW 424	FSFGPRGCLG 448	CYP5418
SL200v200009135	AGHDTS 291	ESLR 350	FRPARF 403	FATGPRQCPA 428	CYP5365
SL200v200009148	AGGDTV 334	EALR 392	FRPQRW 458	FSFGAFACQG 487	Novel
SL200v200009159	AGHDTT 315	ESLR 377	WNPDRF 444	FSAGIRACLG 469	CYP5136
SL200v200009245	TNIDAT 345	ESGR 406	FRPDRF 460	FGMGPRKCLG 480	Novel
SL200v200009596	AGIETT 332	EALR 389	FRPERF 443	FGFGRRICPG 463	CYP5065
SL200v200009604	TNFDGA 313	EAAR 377	YRPDRF 431	FGMGPRKCVG 451	Novel
SL200v200009660	AGSDTT 301	ECLR 358	FNPQRW 411	FGFGRRICPG 432	CYP5152
SL200v200009667	GGADTT 337	ETLR 394	FNPERF 447	FGFGRRICPG 469	CYP5037
SL200v200009815	ASLDTF 319	ETSR 374	FRPERY 427	FGAGRRKCPG 447	CYP504
SL200v200009879	GGTETT 349	EVLR 414	FVPERW 490	FSTGPRGCYG 512	Novel
SL200v200009880	A-GDNV 312	EMLR 390	FIPERF 450	FGFGTTVCPG 477	Novel
SL200v200009900	AASETT 143	ETHR 200	FIPERF 253	FGFGRRVCVG 274	CYP5144
SL200v200009988	GGSETT 308	ESHR 365	FMPERF 418	FGFGRRACPG 439	CYP5144
SL200v200010018	GGADTA 305	ETLR 368	FIPERF 421	FGFGRRVCPG 444	CYP5144
SL200v200010109	AGHDTT 315	EVLR 377	FNPERW 444	FAGGIRSCIG 471	CYP5137
SL200v200010562	TGSDDT 371	ETLR 429	FLPERW 482	FSSGPHACIG 506	CYP5364
SL200v200010592	AGSDTT 347	EALR 412	FWPDRW 469	FSLGPNNCVG 508	CYP5036
SL200v200010597	AGYETT 332	ETLR 388	FNPDRW 455	FADGPRTCLG 482	CYP5340
SL200v200010667	AGSDTT 360	EALR 425	FWPDRW 478	FSLGPNNCVG 517	CYP5036
SL200v200010798	ASLHSS 292	ESQR 348	FDPFRF 402	FGYGKHACPG 432	CYP512
SL200v200011373	SGSETS 234	ETLR 291	YKPERF 344	FGFGRRVCAG 365	CYP5144

Table S3. Sequence motifs for the putative P450s identified in *Serpula lacrymans* var. *shastensis* (SHA) SHA-17. Multiple sequence alignment was carried out using Clustal Omega and sequence position (indicating the position of the first aa) is given alongside. Dashes are used to indicateabsence/incomplete region of a sequence.

**Sequence**	**AGXDTT**	**EXXR**	**PER**	**FXXGXRXCXG**	**Family**
SH17-1v200000116	AAMDTT 326	ETLR 382	WKPERW 449	FLGGGRSCIG 477	CYP5150
SH17-1v200000121	AAMDTT 327	ETLR 383	WKPERW 450	FLGGGRACIG 478	CYP5150
SH17-1v200000144	AGSDTL 305	ETLR 362	FAPERW 416	FSKGPRGCLG 436	CYP5142
SH17-1v200000183	AGSDTI 307	EVHR 363	FRPERF 417	FGFGRRACPG 440	CYP5348
SH17-1v200000265	AGSDTT 346	EELR 401	FRPERW 455	FQFGPFGCLG 479	CYP5143
SH17-1v200001146	GGSDTT 331	ECMR 387	FDPVRF 440	YGFGRRICPG 470	Novel
SH17-1v200001220	TADHTS 296	ETQR 352	FNPFRF 406	FGHGRHACPG 435	CYP512
SH17-1v200001347	AGTDTV 361	ESLR 419	FRPERW 475	FSRGPRSCIG 495	CYP5142
SH17-1v200001348	GGSDTT 298	EILR 355	FNPERF 407	FGYGRRICPG 421	CYP5037
SH17-1v200001442	GGSDTT 385	EVLR 442	FNPDRF 495	FGFGRRICPG 517	CYP5037
SH17-1v200001690	AGHDTT 307	EALR 366	FRPERF 429	FNGGPRRCPA 440	CYP5365
SH17-1v200001691	AGHDTT 294	EALR 353	FRPERF 406	FSSGPRQCPA 427	CYP5365
SH17-1v200001692	AGHDTT 294	ETLR 353	FRPERF 406	FSTGPRQCPA 427	CYP5365
SH17-1v200001693	AGHETT 286	ESLR 345	FRPERF 398	FSSGPRQCPA 418	CYP5365
SH17-1v200001736	AGSDTT 347	EALR 406	FRPERW 461	FSFGPRACVG 481	CYP53
SH17-1v200001870	AGHETT 321	EGVR 377	YRPSRW 442	FSVGPRACIG 478	CYP5154
SH17-1v200001939	AGQHTS 322	ETLR 383	WDPSRW 445	FGAGRHRCIG 490	CYP51
SH17-1v200001954	AGRDNT 310	ETLR 368	FNPARH 429	FGSGPRLCPG 452	CYP5363
SH17-1v200002190	GGVDTI 304	ELFR 361	FRPERF 414	FGYGRRICPG 436	CYP5144
SH17-1v200002278	AGFETT 309	ETMR 366	FFPERH 419	FGFGRRICPG 442	CYP5144
SH17-1v200002293	AGTGTT 303	ELFR 360	FHPERH 413	FGFGRRICPG 434	CYP5144
SH17-1v200002320	AAAETS 302	ETLR 359	FKPERF 412	FGFGRRICVG 433	CYP5144
SH17-1v200002469	AGSDTQ 294	EVLR 351	FNPQRY 404	FGFGRRACPG 420	CYP5747
SH17-1v200002470	GGSHST 315	EVLR 372	FNPDRF 425	FGAGRRICPG 454	CYP5148
SH17-1v200002478	AGHETS 348	ETLR 401	FKPERF 482	FSAGPRACIG 503	CYP5671
SH17-1v200002610	AGHETT 352	ESLR 409	FRPERW 462	FIAGPHACIG 483	CYP5032
SH17-1v200002623	AGHDTT 321	EALR 378	FKPSRF 439	FSAGHRGCVG 456	CYP5141
SH17-1v200002624	AGHETT 321	ETLR 378	FKPSRF 439	FSAGYRGCVG 456	CYP5141
SH17-1v200002631	AGHETT 322	EALR 379	FKPSRF 440	FSAGHRGCVG 457	CYP5141
SH17-1v200002856	AGHDTT 315	ESLR 377	WNPNRF 444	FSAGIRACLG 469	CYP5136
SH17-1v200002865	AGHDTT 292	ESLR 354	WNPNRF 421	FSAGIRACIG 446	CYP5136
SH17-1v200002869	——	ESLR 338	WNPSRF 405	FSAGIRACIG 430	CYP5136

Sequence	AGXDTT	EXXR	PER	FXXGXRXCXG	Family
SH17-1v200002904	GGADTT 293	EVVR 350	FKPERF 403	FGYGRRICPG 422	CYP5037
SH17-1v200002906	GGTDTT 358	EIVR 415	FKPERF 468	FGYGRRICPG 487	CYP5037
SH17-1v200003247	AGRDTT 366	ETLR 423	FDPDRF 483	FNAGPRICLG 507	CYP63
SH17-1v200003297	AGHDTT 315	EVLR 377	FNPERW 444	FAGGIRSCIG 471	CYP5137
SH17-1v200003341	AGAESE 295	EVLR 352	FWPERF 405	FGYGRRICPG 429	CYP5144
SH17-1v200003372	ISIQVL 294	ESLR 350	FDPFRF 423	FGHGKNACPG 452	CYP512
SH17-1v200003395	AGSETT 304	ETLR 361	FKPERF 414	YGFGRRICVG 435	CYP5144
SH17-1v200003683	AGHETS 349	ETLR 412	FKPARF 483	FSAGPRSCIG 504	CYP5671
SH17-1v200003685	VGHETT 316	EGLR 370	FKPERW 427	FCDGPRMCIG 464	CYP5341
SH17-1v200003715	AGHETS 350	ETLR 413	FKPARF 484	FSAGPRGCIG 505	CYP5671
SH17-1v200003823	AGTDTM 315	EGLR 373	FLPDRW 436	FGMGSRACGG 461	CYP5140
SH17-1v200003843	GGSDNT 350	ETLR 480	FLPERW 461	FSSGPHGCIG 485	CYP5364
SH17-1v200003971	AAAETT 322	ETLR 379	FKPERF 432	FGFGRRICVG 452	CYP5144
SH17-1v200003972	AAAETT 302	ETLR 359	FEPDRF 412	FGFGRRICVG 432	CYP5144
SH17-1v200004141	AASETT 334	ETLR 391	FKPERF 444	FGFGRRVCIG 464	CYP5144
SH17-1v200004496	AGSDTT 295	ESFR 352	FQPERW 405	YGFGRRVCAG 426	CYP5152
SH17-1v200004497	AGSDTT 293	ESLR 350	FEPQRW 403	FGFGRRVCPG 424	CYP5152
SH17-1v200004652	AGSDTT 336	EVLR 402	FDPDRW 471	FSAGPRSCIG 496	CYP5151
SH17-1v200004702	AGIETT 331	EALR 368	FRPERF 422	FGFGRRICPG 442	CYP5065
SH17-1v200005054	GAIHTT 297	ETLR 353	FDPFRF 407	FGHGRSACPG 436	CYP512
SH17-1v200005063	AGHDTS 291	ESLR 350	FRPARF 403	FATGPRQCPA 428	CYP5365
SH17-1v200005102	AAIHTS 295	EVLR 351	FDPFRF 405	FGYGRQACPG 435	CYP512
SH17-1v200005177	ALTSNT 285	ESLR 354	FLATRF 514	FGGGISVCSG 436	Novel
SH17-1v200005178	AGSVNT 303	ETIR 371	WNPNRW 423	WGVARFPCTG 444	Novel
SH17-1v200005180	AGRDTT 319	ETLR 376	FDPDRF 436	FNAGPRICLG 460	CYP63
SH17-1v200005190	GGTETT 350	EVLR 415	FVPERW 491	FSTGPRGCYG 513	Novel
SH17-1v200005191	A-GDNV 312	EMLR 390	FIPERF 450	FGFGTTVCPG 477	Novel
SH17-1v200005227	ANVDVS 318	ETLR 372	FKPERF 427	FGFGPRQCLG 447	Novel
SH17-1v200005229	ANIDVT 297	ESAR 355	FRPERF 409	YGLGPRQCLG 429	Novel
SH17-1v200005317	GGMDTN 297	EVFR 354	FNPERY 407	FGFGRRVCPG 427	CYP5037
SH17-1v200005386	GGADTT 446	EVLR 503	FDPERF 556	FGFGRRICPG 578	CYP5037
SH17-1v200005461	GGADTA 305	ETLR 362	FIPERF 415	FGFGRRVCPG 438	CYP5144
SH17-1v200005510	ASIDTT 306	ECLR 363	FKPERY 416	FGFGRRRCPG 441	CYP502
SH17-1v200005648	AGSDTI 303	EVHR 360	FRPERY 414	FGFGRRLCPG 437	CYP5348
SH17-1v200005649	AGSDTI 305	EVHR 362	FRPERF 416	FGFGRRVCPG 439	CYP5348
SH17-1v200005766	TNFDGA 313	EAAR 377	YRPDRF 431	FGMGPRKCVG 451	Novel
SH17-1v200005983	AGHETT 319	EVLR 381	FNPDRW 448	FLGGIRSCIG 474	CYP5137
SH17-1v200006150	AGADT- 294	ETCR 354	FDPQRW 456	FGFGRRVCPG 477	Novel

SH17-1v200006151	AGFDTT 293	EALR 368	FDPGRW 421	FGFGRRVCPG 442	CYP5152
SH17-1v200006177	AGSDTS 296	ETLR 353	FNPARW 406	FGFGRRSCPG 427	CYP5152
SH17-1v200006178	AGSDTT 296	ETLR 353	FNPQRW 406	FGFGRRACPG 427	CYP5152
SH17-1v200006209	AGADTI 373	ESFR 430	FNPFRW 483	YGFGQRACPG 504	CYP5152
SH17-1v200006223	AGHETT 345	ETLR 400	FKPERW 467	FLGGPRACIG 492	CYP5139
SH17-1v200006455	SGSETS 303	ETLR 360	YRPERF 413	FGFGRRICVG 434	CYP5144
SH17-1v200006467	AGYEPV 335	ETIR 389	FRPERW 453	FSFGPRGCLG 477	CYP5418
SH17-1v200006475	AGSDST 347	ETLR 408	FNPDRW 478	FSFGPHACPG 504	CYP5151
SH17-1v200006722	AGSDTT 293	ESFR 350	FEPQRW 403	YGFGRRACPG 424	CYP5152
SH17-1v200006782	GGVETT 262	ETLR 319	FDPSRF 372	FGSGRRICPG 393	Novel
SH17-1v200006796	AGHETT 334	ETLR 390	FRPERW 457	FLSGSRGCIG 482	CYP5138
SH17-1v200006993	AGGDTV 334	EALR 392	FRPQRW 458	FSFGAFACQG 487	Novel
SH17-1v200007220	SAIHTS 296	ETLR 352	FDPFRF 406	FGHGKNSCPG 435	CYP512
SH17-1v200007325	——	ESIR 229	FDPMRY 318	FGYGRLKCVA 335	CYP5153
SH17-1v200007331	ATIHTL 321	ESLR 377	FDGFRF 431	FGYGSLACPG 457	Novel
SH17-1v200007786	AGSDTT 370	ETLR 427	FDPDRF 486	FNAGPRICLG 510	CYP63
SH17-1v200007787	AGRDTT 367	ETLR 424	FDPDRF 478	FNAGPRICLG 502	CYP63
SH17-1v200007996	AGSDTT 298	ECLR 355	FNPQRW 408	FGFGRRICPG 429	CYP5152
SH17-1v200008080	AASESS 305	ETLR 362	FRPERF 415	FGFGRRNCVG 436	CYP5144
SH17-1v200008321	AGYETT 330	EVLR 385	FNPERW 452	FSDGPRTCLG 479	CYP5340
SH17-1v200008322	AGYETT 336	ETLR 392	FNPDRW 454	FADGPRTCLG 481	CYP5340
SH17-1v200008342	AGARAT 293	ETLR 367	FDPGRK 419	WGGGKTICLG 436	CYP5156
SH17-1v200008432	AGSDTT 346	EALR 411	FWPDRW 468	FSLGPNNCVG 507	CYP5036
SH17-1v200008442	AGSDTT 373	ESFR 430	FDPRRW 483	FGFGRRACPG 504	CYP5152
SH17-1v200008534	AGSDTT 343	ETLR 401	FWPERW 458	FSFGPANCVG 495	CYP5035
SH17-1v200008535	AGSDTT 349	EALR 408	FWPERW 465	FSFGPAMCVG 503	CYP5035
SH17-1v200008538	AGHDTT 315	ESLR 377	WNPNRF 444	FSAGIRACIG 469	CYP5136
SH17-1v200008590	ASQDAM 311	ESMR 370	LTPERW 423	FGSGPHKCIG 445	CYP61
SH17-1v200008641	AGHETT 318	ETLR 380	FRPERW 447	FGSGPHACIG 474	CYP5137
SH17-1v200008681	AGHETS 288	ETMR 345	FEPSRW 408	FSHGTRACIG 426	Novel
SH17-1v200008702	GGADTI 299	EVLR 355	FKPERH 408	FGFGRRICPG 428	CYP5352
SH17-1v200008740	ASLDTF 319	ETSR 374	FRPERY 427	FGAGRRKCPG 448	CYP504
SH17-1v200008782	GGADTT 298	ETLR 355	FKPERF 408	FGFGRRICPG 430	CYP5037
SH17-1v200008802	GGADTT 330	EVLR 387	FNPSRF 440	FGFGRRICPG 462	CYP5037
SH17-1v200008840	TAIHSS 297	ETLR 353	FDPFRF 407	FGHGRGSCPG 436	CYP512
SH17-1v200008906	AGHETT 325	EGVR 381	YRPSRW 446	FSVGPRACIG 476	CYP5154
SH17-1v200009038	GGTETT 302	ELHR 359	FKPERF 411	FGFGRRRCPG 431	CYP5149
SH17-1v200009190	——	EALR 924	FDASRS 976	RGDGAFKCLG 988	CYP6005
SH17-1v200009260	AGRDTT 363	ETLR 420	FDPDRF 480	FNAGPRICLG 504	CYP63
SH17-1v200009607	GASETT 324	ETQR 381	FNPQRF 434	FGFGRRICVG 455	CYP5144

SH17-1v200009616	GATETT 307	ESHR 364	FVPERF 417	FGFGRRVCAG 438	CYP5144
SH17-1v200009619	GGSETT 308	ESHR 365	FMPERF 415	FGFGRRACPG 436	CYP5144
SH17-1v200009718	AASETS 302	ETLR 359	FKPERF 412	FGFGRRVCVG 433	CYP5144
SH17-1v200009719	AAAETS 307	ETLR 364	FKPERF 417	FGFGRRICVG 438	CYP5144
SH17-1v200009777	AAAETT 330	ETHR 387	FIPERF 440	FGFGRRVCVG 461	CYP5144
SH17-1v200010294	TNIDAT 155	ESGR 216	FRPERF 270	FGMGPRKCLG 290	Novel
SH17-1v200010737	GATETT 310	ESHR 367	FVPERF 420	FGFGRRVCAG 441	CYP5144
SH17-1v200011276	AAIHTS 296	EAQR 352	FQPFRF 406	FGHGRHACPG 436	CYP512
SH17-1v200011367	ASLHSS 292	ESQR 348	FDPFRF 402	FGYGKHACPG 432	CYP512
SH17-1v200011565	AGHETT 316	EALR 373	FKPSRW 436	FGHGPRVCIG 454	CYP5154
SH17-1v200011895	AGTDTV 266	EVHR 323	FRPERF 377	FGFGRRVCSG 400	CYP5348

Table S4. Sequence motifs for the putative P450s identified in *Serpula himantioides* (SH) MUCL38935. Multiple sequence alignment was carried out using Clustal Omega and sequence position (indicating the position of the first aa) is given alongside. Dashes are used to indicate absence/incomplete region of a sequence. * indicates the two halves of the large fusion protein MUCL38935v200007922.

**Sequence**	**AGXDTT**	**EXXR**	**PER**	**FXXGXRXCXG**	**Family**
MUCL38935v200000177	ASLHSS 293	ESQR 350	FDPFRF 403	FGYGKHACPG 433	CYP512
MUCL38935v200000644	AGSDST 349	ETLR 413	FNPDRW 483	FSFGPHACPG 509	CYP5151
MUCL38935v200000652	AGYEPV 335	ETIR 389	FRPERW 453	FSFGPRGCLG 477	CYP5418
MUCL38935v200000866	HSNDQL 641	EALR 701	FDASRS 753	WGDGAFKCLG 765	CYP6005
MUCL38935v200001039	AGHETS 340	ETLR 396	FRPERW 463	FLSGSRGCIG 488	CYP5138
MUCL38935v200001199	AGSETT 326	ETLR 383	FXPERF 436	FGFGRRICVG 457	CYP5144
MUCL38935v200001391	AGHETT 318	ETLR 380	FRPERW 447	FGSGPHACIG 474	CYP5137
MUCL38935v200001461	ASIHQT 296	EVMR 358	FRPERW 411	FGLGRWACPG 434	CYP5347
MUCL38935v200001579	AGSETT 346	ETLR 403	FKPERF 456	FGFGRRVCVG 476	CYP5144
MUCL38935v200001717	ASLDTF 319	ETSR 374	FRPERY 427	FGAGRRKCPG 448	CYP504
MUCL38935v200001850	AGSDTT 307	ESFR 364	FDPRRW 417	YGFGRRACPG 438	CYP5152
MUCL38935v200001953	ASIDTT 304	ECLR 361	FKPERY 414	FGFGRRRCPG 439	CYP502
MUCL38935v200002310	TNFDGT 313	EAAR 377	YRPDRF 431	FGMGPRKCVG 451	Novel
MUCL38935v200002399	AAAETT 295	ETLR 352	FKPERF 405	FGFGRRICIG 425	CYP5144
MUCL38935v200002400	AAAEST 304	ETFR 361	FKPERF 414	FGFGRRICVG 434	CYP5144
MUCL38935v200002401	AAAETT 304	ETLR 361	FEPDRF 414	FGFGRRICVG 434	CYP5144
MUCL38935v200002461	GGADTI 299	EVLR 355	FNPERH 408	FGFGRRICPG 428	CYP5352
MUCL38935v200002560	AATETS 305	ETLR 362	FEPERF 415	FGFGRRICVG 436	CYP5144
MUCL38935v200002635	AGHETT 317	EVLR 379	FNPERW 446	FIGGTRACIG 472	CYP5137
MUCL38935v200002950	AASETT 306	ELYR 363	FIPERF 416	FGFGRRVCVG 437	CYP5144
MUCL38935v200003212	AGSDTV 305	EVHR 362	FRPERF 416	FGFGRRVCPG 439	CYP5348
MUCL38935v200003268	AGHETT 327	ETLR 382	FKPERW 449	FLGGPRACIG 474	CYP5139
MUCL38935v200003291	QGSDTS 306	EVHR 363	FIPERF 416	FGSGRRVCPG 445	CYP5416
MUCL38935v200003294	AGSDTL 134	ETLR 192	FVPERW 263	FSRGPRACLG 284	CYP5142
MUCL38935v200003313	DAN— 181	ETMR 233	FDPLRF 287	FGLGKHACPG 317	CYP512
MUCL38935v200003315	AGADTT 307	ETHR 364	FMPERF 417	FGFGRRVCPG 439	CYP5144
MUCL38935v200003469	AGSDTT 346	EALR 411	FWPDRW 468	FSLGPNNCVG 508	CYP5036
MUCL38935v200003639	AASESS 303	ETLR 360	FRPERF 413	FGFGRRNCVG 435	CYP5144
MUCL38935v200003787	VGHETT 316	EGLR 370	FKPERW 437	FCDGPRMCIG 464	CYP5139
MUCL38935v200003948	AGYETT 330	EILR 385	FNPERW 452	FSDGPRTCLG 480	CYP5340
MUCL38935v200003949	GGSDTT 298	EVLR 355	FNPERF 407	FGYGRRICPG 430	CYP5037

MUCL38935v200003950	AGTDTV 308	ESLR 366	FRPERW 422	FSRGPRSCIG 443	CYP5142
MUCL38935v200003995	AGADTT 294	ETLR 351	FDPQRW 404	FGFGRRVCPG 426	CYP5152
MUCL38935v200003996	AGFDTT 293	EALR 250	FDPGRW 403	FGYGRRVCPG 425	CYP5152
MUCL38935v200004051	CGADTT 303	EVVR 360	FKPERF 413	FGYGRRICPG 433	CYP5037
MUCL38935v200004052	GGTDTT 298	EVVR 355	FKPERF 408	FGYGRRICPG 428	CYP5037
MUCL38935v200004057	AGADTT 296	EVVR 353	FKPERF 406	FGYGRRICPG 426	CYP5037
MUCL38935v200004058	AGSDTT 301	ETLR 358	FKPERF 411	FGFGRRVCVG 433	CYP5144
MUCL38935v200004104	AGHETT 317	ETLR 379	WNPTRF 446	FSAGVRACIG 472	CYP5136
MUCL38935v200004205	GGADTT 298	EVLR 355	FNPDRF 408	FGFGRRICPG 431	CYP5037
MUCL38935v200004206	GGADTT 298	EVLR 355	FNPDRF 408	FGFGRRICPG 431	CYP5037
MUCL38935v200004236	AAMDTT 325	ETLR 382	WKPERW 449	FLGGGRSCIG 478	CYP5150
MUCL38935v200004319	AGARAT 293	ETLR 367	FDPGRK 419	WGGGKTICLG 437	CYP5156
MUCL38935v200004390	AGHETT 325	EGVR 381	YRPSRW 446	FSVGPRACIG 475	CYP5154
MUCL38935v200004477	AASETT 351	ETLR 408	FRPERF 461	FGFGRRXCXD 483	CYP5144
MUCL38935v200004483	AGSDTT 347	EALR 406	FWPXRW 463	FSFGPAMCVG 502	CYP5035
MUCL38935v200004562	GGADTT 298	ETLR 355	FRPERF 408	FGFGRRICPG 431	CYP5037
MUCL38935v200004699	AGRDTT 366	ETLR 423	FDPXRF 483	XNAGPRICLG 508	CYP63
MUCL38935v200004767	AGADTM 389	ESFR 445	FDPLRW 498	YGFGQRVCPG 520	CYP5152
MUCL38935v200004827	AASDTT 309	ETLR 366	FKPERF 419	FGFGRRVCVG 440	CYP5144
MUCL38935v200005015	AGQHTS 322	EXLR 383	WDPSRW 445	FGAGRHRCIG 491	CYP51
MUCL38935v200005022	GGSDTT 328	ECMR 385	FDPTRF 438	YGFGRRICPG 469	Novel
MUCL38935v200005040	AAAETT 139	ETLR 196	FIPDRF 249	FGFGRRICVG 270	CYP5144
MUCL38935v200005106	AGHDTT 315	EVLR 368	FNPDRW 444	FAGGIRSCIG 472	CYP5137
MUCL38935v200005207	AGRDTT 367	ETLR 424	FDPDRF 478	FNAGPRICLX 503	CYP63
MUCL38935v200005208	AGGDTT 369	ETLR 426	FDPDRF 485	FNAGPRICLG 510	CYP63
MUCL38935v200005233	PAFETM 211	EVLR 265	FDPMRY 320	FGFGRLKCIA 338	CYP5153
MUCL38935v200005321	AGYETT 330	EILR 385	FNPERW 452	FSDGPRTCLG 480	CYP5340
MUCL38935v200005372	AGHDTT 285	EALR 344	FRPERF 370	FSSGPRRCPA 419	CYP5365
MUCL38935v200005634	AAVETS 306	ETLR 363	FKPERF 416	FGFGRRICVG 438	CYP5144
MUCL38935v200005681	AAMDTT 331	ETLR 387	WKPERW 454	FSGGGRSCIG 483	CYP5150
MUCL38935v200005684	AGSETT 328	ESLR 383	FRPERW 449	FSDGPRTCVG 475	CYP5341
MUCL38935v200005700	AAAETS 121	ETLR 178	FKPERF 231	FGFGRRVCVG 253	CYP5144
MUCL38935v200005702	AGSETS 303	ETLR 360	YKPERF 413	FGFGRRICVG 435	CYP5144
MUCL38935v200005720	AGHDTS 291	ESLR 350	FRPTXF 403	FATGPRQCPA 429	CYP5365
MUCL38935v200005779	AGTDTT 325	EVFR 384	FMPERF 437	FGFGRRKCPG 459	CYP5065
MUCL38935v200005915	AGHETT 339	EVLR 401	FDPERW 468	FLSGIRSCIG 495	CYP5137
MUCL38935v200005935	AAAETS 320	ETLR 377	FKPERF 430	FGFGRRICVG 452	CYP5144

MUCL38935v200005936	GGAETT 278	ETLR 335	FKPERF 388	FGFGRRICPG 410	CYP5144
MUCL38935v200005995	AGSDTT 293	ESFR 350	FEPQRW 403	YGFGRRACPG 425	CYP5152
MUCL38935v200006039	AGHETT 319	EVLR 381	FNPDRW 448	FLGGVRSCIG 475	CYP5137
MUCL38935v200006111	AGRDTT 364	ETLR 421	FDPDRF 476	FNAGPRICLG 501	CYP63
MUCL38935v200006196	GGADTT 298	EVLR 355	FNPXRF 408	FGFGRRICPG 431	CYP5037
MUCL38935v200006275	AAIHTS 296	EAQR 352	FDPFRF 406	FGHGRHACPG 437	CYP512
MUCL38935v200006276	AAIHTT 296	EAQR 352	FXPFRF 406	FGHGRHACPG 437	CYP512
MUCL38935v200006540	TNIDAT 352	ESGR 413	FRPERF 467	FGMGPRKCLG 488	Novel
MUCL38935v200006550	AGSDTT 291	ETFR 348	FDPQRW 401	YGFGRRVCPG 423	CYP5152
MUCL38935v200006609	AGSEPT 313	ETLR 370	FIPERF 423	FGFGRRVCVG 445	CYP5144
MUCL38935v200006661	AGHETT 321	EALR 378	FKPSRF 439	FSAGYRGCVG 457	CYP5141
MUCL38935v200006662	AGHETT 323	ETLR 380	FKPSRF 441	FSAGHRGCVG 459	CYP5141
MUCL38935v200006670	AGSDTT 99	EELR 157	FRPERW 211	FQFGPFGCLG 236	CYP5143
MUCL38935v200006673	AGSDTS 347	ETLR 404	FNPGRW 457	FGFGRRACPG 479	CYP5152
MUCL38935v200006728	AGSDTS 296	ETLR 353	FNPERW 406	FGFGRRSCPG 428	CYP5152
MUCL38935v200006929	AGSDTT 347	EALR 406	FRPERW 461	FSFGPRACVG 482	CYP53
MUCL38935v200007161	GGSDNT 317	ETLR 375	FLPERW 428	FSSGPHGCIG 453	CYP5364
MUCL38935v200007174	AAVGTL 295	ETLR 352	FRPERF 405	FGFGRRVCVG 427	CYP5144
MUCL38935v200007324	AGHETT 296	EVLR 358	FNPDRW 425	FLGGTRACVG 452	CYP5137
MUCL38935v200007393	GASETT 305	ESHR 362	FIXERF 415	FGFGRRICIG 437	CYP5144
MUCL38935v200007394	AAAETT 306	ETHR 363	FIPERF 416	FGFGRRVCVG 438	CYP5144
MUCL38935v200007459	GGNDTT 329	ESIR 385	YRPSRW 450	FGIGSRACIG 485	CYP5154
MUCL38935v200007461	TGHETT 296	ESVR 352	YRPSRW 417	FSVGPRTCIG 452	CYP5154
MUCL38935v200007614	AXXETS 349	ETLR 411	FKPERF 482	FSAGPRACIG 504	CYP5671
MUCL38935v200007675	AARDTT 364	ETLR 420	FDPERW 486	FSAGPRICIG 511	CYP63
MUCL38935v200007752	AGLETT 296	EIYR 353	FNPSRF 406	FGFGRRICPG 428	CYP5144
MUCL38935v200007922a*	AGADST 198	ETLR 255	FKPERH 308	FGFGRRICPG 331	CYP5144
MUCL38935v200007922b*	AGFETT 883	ETMR 940	FLPERH 993	FGFGRRICPG 1017	CYP5144
MUCL38935v200007938	ASRDNT 310	ETLR 368	FNPARH 429	FGSGPRLCPG 453	CYP5363
MUCL38935v200007973	AGSDTS 295	ETLR 352	FDPGRW 405	FGFGRRSCPG 427	CYP5152
MUCL38935v200007990	AGYETT 331	ETLR 387	FKPERW 454	FADGPRTCLG 482	CYP5340
MUCL38935v200008101	TGTITT 288	ETNR 358	WDPARW 410	WGVGLHPCLG 434	Novel
MUCL38935v200008111	TANSDS 227	ETLR 283	FEPFRF 337	FGLGRHACPG 437	CYP512
MUCL38935v200008267	AGHETT 316	EAMR 373	FKPSRW 436	FGHGPRVCIG 455	Novel
MUCL38935v200008268	IGTAPS 299	ETVR 369	WNPARW 421	WGIGMHPCLG 445	Novel
MUCL38935v200008271	IGTNTT 289	ETVR 359	WDPARW 411	WGAGLHPCHG 435	Novel

MUCL38935v200008329	AGSDTT 294	ESLR 351	FEPGRW 404	YGFGRRXCPG 426	CYP5152
MUCL38935v200008330	AGSDTT 295	ESFR 352	FXPERW 405	YGFGRRVCAG 427	CYP5152
MUCL38935v200008376	AGAESE 314	EVLR 371	FWPERF 424	FGYGRRMCPG 449	CYP5144
MUCL38935v200008382	AGRDTT 364	ETLR 421	YDPDRF 481	FNAGPRICLG 506	CYP63
MUCL38935v200008433	GGADPL 282	ESLR 339	FNPLRW 392	YGFGQRVCPG 414	CYP5152
MUCL38935v200008459	AGHETS 320	ETMR 377	FEPSRW 440	FSHGTRACIG 459	Novel
MUCL38935v200008560	ATIHTS 320	EVLR 376	FDPFRF 430	FGYGRHACPG 461	CYP512
MUCL38935v200008574	SAI— 297	ETLR 349	FDPFRF 403	FGHGRNACPG 433	CYP512
MUCL38935v200008750	IGI— 298	ETQR 350	FTPFRF 404	FGHGENACPG 434	CYP512
MUCL38935v200008783	AGHDTT 296	ESLR 355	FRPERF 408	FSGGPRRCPA 430	CYP5365
MUCL38935v200008784	AGHDTT 294	EALR 353	FRPERF 406	FSTGPRQCPA 428	CYP5365
MUCL38935v200008787	AGHETT 286	ESLR 345	FRPERF 398	FASGPRQCPA 419	CYP5365
MUCL38935v200008892	GXAETT 330	ETLR 387	FNPERY 440	FGFGRRICPG 463	CYP5144
MUCL38935v200008893	AGTGTA 466	EIFR 523	FDPERH 576	FGFGRRICPG 598	CYP5144
MUCL38935v200009064	ASQDAM 323	ESLR 382	LTPERW 435	FGSGPHKCIG 458	CYP61
MUCL38935v200009081	AGSDTT 365	ETLR 423	FWPERW 480	FSFGPANCVG 518	CYP5035
MUCL38935v200009217	AAIDTS 295	ECFR 351	FDPYRF 405	FGHGKHACPG 436	CYP512
MUCL38935v200009354	AGIETX 248	EALR 305	FRPERF 359	FGFGRRICPG 380	CYP5065
MUCL38935v200009457	GASETT 305	ESHR 362	FKPERF 415	FGFGRRICVG 437	CYP5144
MUCL38935v200009512	AGTDTM 315	EGLR 373	FLPDRW 436	FGTGSRACGG 462	CYP5140
MUCL38935v200009531	IGTVTT 293	ETVR 363	WDPARW 415	WGIGLHPCHG 439	Novel
MUCL38935v200009532	AAAETT 129	ETHR 186	FIPERF 239	FGFGRRXCVG 261	CYP5144
MUCL38935v200009755	AAMDTT 180	ETLR 237	WKPERW 304	FLGGNRACIG 333	CYP5150
MUCL38935v200010217	AGHETT 317	EVLR 379	FNPDRW 446	FSGGVRSCIG 473	CYP5137
MUCL38935v200010266	ANIDVT 327	ESAR 385	FRPERF 439	YGLGPRQCLG 460	Novel
MUCL38935v200010269	AGYETT 331	ETLR 387	FKPERW 454	FADGPRTCLG 482	CYP5340
MUCL38935v200010711	AGRDNV 303	ETLR 361	FDPGRY 422	FGAGPRLCPG 446	CYP5363
MUCL38935v200010826	GGADTI 299	EVLR 355	FNPERH 408	FGFGRRICPG 429	CYP5352
MUCL38935v200011081	GGSDST 308	ESLR 365	FLPERY 418	FGFGRRRCPG 443	CYP5351
MUCL38935v200011232	FAGDNV 301	EMLR 380	FIPERF 440	FRFGTTVCPG 468	Novel
MUCL38935v200011268	AGSDTT 293	ESLR 350	FEPQRW 403	FGFGRRVCPG 425	CYP5152
MUCL38935v200011477	AGHETT 286	ESLR 345	FRPERF 398	FASGPRQCPA 419	CYP5365
MUCL38935v200011647	AAAETT 235	ETHR 292	FIPERF 345	FGFGRRXCVG 367	CYP5144
MUCL38935v200011704	AGSDTT 293	ESLR 350	FDPQRW 403	YGFGRRVCPG 425	CYP5152
MUCL38935v200011739	AGSDTT 293	ESLR 353	FEPQRW 406	FGFGRRVCPG 428	CYP5152
MUCL38935v200011938	AGGEST 314	EAMR 371	FVPERH 424	FGFGRRICPG 447	CYP5158

MUCL38935v200012398	GGLETS 307	ELFR 364	FDPARY 417	FGFGRRACPG 438	CYP5350
MUCL38935v200012461	AGADTI 113	ESFR 170	FNPLRW 223	YGFGKRVCPG 245	CYP5152
MUCL38935v200012513	AGYEXT 331	ETLR 387	FKPERW 454	FADGPRTCLG 482	CYP5340

Table S5. The number of significantly up-regulated family members seen in the core wood transcriptome in each strain.

Strain	Family	Number of different family members expressed
SLL	5152	2
	5037	2
SHA	63	3
SH	5137	4
	5144	5
	63	3
	5340	2
	5152	2
